# Gut Microbiota and Metabolic Dysfunction-Associated Steatotic Liver Disease

**DOI:** 10.3390/antiox13111386

**Published:** 2024-11-14

**Authors:** Emidio Scarpellini, Marialaura Scarcella, Jan F. Tack, Giuseppe Guido Maria Scarlata, Michela Zanetti, Ludovico Abenavoli

**Affiliations:** 1 Translational Research in Gastroeintestinal Disorders, Gasthuisberg University Hospital, KULeuven, Herestraat 49, 3000 Lueven, Belgium; jan.tack@kuleuven.be; 2 Anesthesia, Intensive Care and Nutritional Science-Azienda Ospedaliera “Santa Maria”, Via Tristano di Joannuccio, 05100 Terni, Italy; m.scarcella@aospterni.it; 3 Department of Health Sciences, University “Magna Graecia”, 88100 Catanzaro, Italy; giuseppeguidomaria.scarlata@unicz.it (G.G.M.S.); l.abenavoli@unicz.it (L.A.); 4 Geriatrics Department, Nutrition and Malnutrition Unit, Azienda Sanitario-Universitaria Giuliano Isontina, Ospedale Maggiore, piazza dell’Ospitale 1, 34100 Triste, Italy; michela.zanetti@asugi.sanita.fvg.it

**Keywords:** gut microbiota, dysbiosis, gut–liver axis, probiotics, liver steatosis, oxidative stress

## Abstract

**Background:** The gut microbiota constitutes a complex microorganism community that harbors bacteria, viruses, fungi, protozoa, and archaea. The human gut bacterial microbiota has been extensively proven to participate in human metabolism, immunity, and nutrient absorption. Its imbalance, namely “dysbiosis”, has been linked to disordered metabolism. Metabolic dysfunction-associated steatotic liver disease (MASLD) is one of the features of deranged human metabolism and is the leading cause of liver cirrhosis and hepatocellular carcinoma. Thus, there is a pathophysiological link between gut dysbiosis and MASLD. **Aims and Methods:** We aimed to review the literature data on the composition of the human bacterial gut microbiota and its dysbiosis in MASLD and describe the concept of the “gut–liver axis”. Moreover, we reviewed the approaches for gut microbiota modulation in MASLD treatment. **Results:** There is consolidated evidence of particular gut dysbiosis associated with MASLD and its stages. The model explaining the relationship between gut microbiota and the liver has a bidirectional organization, explaining the physiopathology of MASLD. Oxidative stress is one of the keystones in the pathophysiology of MASLD and fibrosis generation. There is promising and consolidated evidence for the efficacy of pre- and probiotics in reversing gut dysbiosis in MASLD patients, with therapeutic effects. Few yet encouraging data on fecal microbiota transplantation (FMT) in MASLD are available in the literature. **Conclusions:** The gut dysbiosis characteristic of MASLD is a key target in its reversal and treatment via diet, pre/probiotics, and FMT treatment. Oxidative stress modulation remains a promising target for MASLD treatment, prevention, and reversal.

## 1. Introduction

Metabolic dysfunction-associated steatotic liver disease (MASLD) can be considered a hepatic feature of metabolic syndrome [1]. Histologically, MASLD is defined by the presence of hepatic steatosis in up to about 5–30% of liver biopsies performed on patients without heavy alcohol consumption or other causes of chronic liver disease (e.g., viral, alcohol, drugs, primary hepatic disease, etc.) [2]. Indeed, the features of MASLD range from steatosis to non-alcoholic steatohepatitis (namely NASH), characterized by liver fibrosis development until cirrhosis and hepatocellular carcinoma [3]. The change in nomenclature from NAFLD to MASLD took place in 2023, following an accurate Delphi process and multisocietary consensus [4]. In detail, the term “non-alcoholic” from NAFLD does not include the etiology of the disease. Moreover, there are patients with risk factors for NAFLD (e.g., type 2 diabetes or moderate alcohol consumption) excluded from clinical trials and disease treatment. In addition, there is an overlap of pathophysiological processes that contribute to both NAFLD and alcohol-associated/related liver disease (ALD). Thus, in 2020, Eslam et al. proposed the use of the term metabolic dysfunction-associated fatty liver disease (MAFLD). The latter includes patients with a fatty liver, regardless of the amount and pattern of alcohol intake [5]. However, there have been concerns about the group of etiologies included in the definition. Further, there are concerns about the use of the term “fatty”, which could restrict the population to those with two metabolic risk factors and with more liberal alcohol use. Altogether, these changes in diagnostic criteria could have a negative impact on the diseases’ biomarkers and future therapeutic approaches. Thus, the new MASLD nomenclature includes the presence of at least one out of five cardiometabolic risk factors. Therefore, a new definition of disease, termed metabolic and alcohol-related/associated liver disease (MetALD), was introduced to describe patients with MASLD who consume greater amounts of alcohol per week (140–350 g/week and 210–420 g/week for females and males, respectively) [4]. The new definition overcomes the stigma of the term “fatty”, differentiates moderate use from the abuse of alcohol, and includes the pathophysiologic dysmetabolic milieu of the disease.

The worldwide prevalence of MASLD has grown to 37.3% of the general population [3]. The pathophysiology of MASLD is complex and multifaceted. All the stages of MASLD recognize, as the first step, the excessive accumulation of lipids in the liver. The latter can depend on the consumption of a high-calorie, high-fat diet (namely a Westernized diet) and increased lysis of adipose tissue and the de novo hepatic genesis of adipose tissue [6]. Other etio-pathogenetic factors are genetic and epigenetic, metabolic (e.g., insulin resistance and lipidic profile toxicity), oxidative stress and mitochondrial dysfunction, and finally, the derangement of the microbiota–gut–liver axis [7].

Interestingly, MASLD patients exhibit a typical “dysbiosis” (namely qualitative and quantitative change) in their gut microbiota, which is caused by increased gut permeability and the translocation of intestinal bacterial antigens to the liver via the enterohepatic circulation [8,9].

The composition of the gut microbiota is influenced by geographical, genetic, dietary, and lifestyle factors [10]. Thus, there is a need for efficient and non-time-consuming methods that are able to analyze variations in microbiota composition. In this regard, newer non-culture-based techniques such as 16S-rRNA sequencing and metagenomic methods allow for high-accuracy approaches to reveal changes in the structure of the gut microbiota. They belong to the “targeted” and “non-targeted” methods, respectively. Targeted versions can detect specific sequences (namely the target sequence) in the gene by using targeted amplification and detection. On the other hand, non-targeted sequencing can be used to study entire genome sequencing, which accurately distinguishes the genetic differences between strains but costs time and energy. Thus, new-generation metagenomic sequencing can be used to obtain all genetic information for the reconstruction of gut microbiota taxa starting directly from the sample under examination [11]. However, despite the promising and efficient methods of studying the gut microbiota, there is still a lot of research being conducted to study and define the precise interaction between gut dysbiosis and MASLD pathophysiology.

Oxidative stress has a relevant role in the gut–liver axis. It has been described in drug-induced liver injury (DILI) [12], toxicant-associated fatty liver disease (TAFLD) [13], alcohol-associated liver disease (ALD) [14], and mostly in MASLD [15]; in fact, mitochondria, endoplasmic reticula (ER), and peroxisomes are damaged and become dysfunctional. Therefore, the overproduction of reactive oxygen species (ROS) occurs [16,17]. This starts a vicious cycle [15], and as a result, oxidative stress causes DNA oxidation, lipid peroxidation, oxidative protein alterations, the derangement of fat metabolism, systemic inflammation, and tissue damage [18,19]. Altogether, these damages catalyze the progression of liver diseases of any origin [20].

On the other hand, hepatocytes react to oxidative stress in a sophisticated way: hepatic stellate cells (HSCs) activated by ROS and damage-associated molecular patterns (DAMPs) derived from damaged hepatocytes will contribute to building the extracellular matrix that composes fibrotic tissue [18]. Oppositely, Kupffer cells are activated by endotoxins (namely lipopolysaccharides (LPSs)) or superoxide anions and will contribute to ROS production through the stimulation of NADPH-dependent oxidases (NOXs) and the redox-sensitive transcription factor nuclear factor-κB (NF-κB)-mediated pro-inflammatory storm of cytokines, chemokines, and cell adhesion molecules (CAMs) [18]. As a final step, hepatocytes will respond through necrotic and apoptotic pathways.

Thus, we reviewed data from scientific literature on the composition of healthy gut microbiota and on gut dysbiosis in MASLD patients with a focus on the gut–liver axis concept and oxidative stress. Finally, we reviewed the evidence on gut microbiota modulation for MASLD treatment.

## 2. Materials and Methods

We conducted a search on PubMed and Medline for literature data (namely original articles, reviews, meta-analyses, and case series) using the following keywords, their acronyms, and their associations (e.g., “and”): gut microbiota, dysbiosis, gut–liver axis, probiotics, liver steatosis, and oxidative stress. We chose these keywords because they are the most representative of the relationship between the gut microbiota, liver steatosis, and oxidative stress pathophysiological link. Moreover, preliminary evidence from abstracts from the main national and international hepatological and gastroenterological meetings (e.g., the Italian association for liver disease study, the European association for the study of liver disease, United European Gastroenterology Week, and Digestive Disease Week) was also included. The articles found in the search were reviewed by two of the authors (E.S. and L.A). The last Medline search was operated on 31 July 2024.

## 3. Results

### 3.1. Gut Microbiota and MASLD

#### 3.1.1. Gut Microbiota “Eubiosis”

The gut microbiota harbors the superficial mucous layer of intestinal mucosa (namely the “permanent“ gut microbiota) but most bacteria harbor the stool (namely “transient“ gut microbiota) [21]. The peristaltic intestinal interdigestive and digestive motility (regulated by the enteric nervous system), gastric juice, pancreatic and biliary secretions, the enterocytes’ life cycle, the immune system, and the liver modulate the gut microbiota composition during the lifespan [22].

The microbiota is already present in the gut during the prenatal period [23,24]. At this age, the microbes’ composition is affected by placental function, maternal health status, and diet. Furthermore, the type of delivery (namely vaginal vs. caesarean), breastfeeding vs. formula feeding, geographical differences, environmental hygiene, and, finally, interactions with family members are all factors conditioning the gut microbiota composition in infancy and childhood [13]. The gut microbiota will have an increasing diversity up to the reaching of three years of age. Afterwords, the microbiota’s composition will stabilize, reaching the adulthood makeup [25].

Interestingly, adults’ gut microbial composition is mainly affected by environmental factors rather than host genetics. Among the various factors, we can include age, diet, physical activity, gastrointestinal motility and secretive functions (specifically GI tract pH and oxidative status), the use of acid suppressive treatments, the use and misuse of antibiotics, and toxic substances within the environment [14,26].

In detail, two out of twelve phyla of the adult human bacterial gut microbiota are considered major: the Gram-positive bacteria *Firmicutes* (60% of the whole bacterial gut microbiota), with their two main classes represented by *Bacilli* (ranging from obligate to facultative aerobes) and *Clostridia* (anaerobic), and *Bacteroidetes* (10% of the whole bacterial gut microbiota), which are Gram-negative anaerobic bacteria. Further, *Actinobacteria*, *Proteobacteria*, *Fusobacteria*, and *Verrucomicrobia (Akkermansia)* are less represented phyla [14,15].

The gut microbiota dynamically interacts with the intestinal mucosa, whose layer is made up of enterocytes. Enterocytes participate in the absorption of nutrients (e.g., sugars, lipids, peptides, and amino acids), ions, water, and vitamins, and, importantly, they regulate transcellular and intestinal permeability. In addition, the intestinal mucosa also includes goblet cells that release mucin, tuft cells with chemo-sensing and immune-surveillance functions, and enteroendocrine cells that produce hormones that regulate GI motility, hunger, appetite, and, finally, gut microbiota composition. Among these, we list motilin, cholecystokinin, glucagon-like peptide 1 (GLP-1), GLP-2, peptide YY (PYY), vasoactive intestinal peptide (VIP), and fibroblast growth factor 19 (FGF-19). The intestinal wall recognizes as an immune effector the lymphoid tissue associated with the mucosa (MALT), whose main actors are the Microfold (“M”) cells that sample luminal antigens for subsequent presentation [27].

Typically, enteric cells connect to each other through tight junctions, adherens junctions, and, finally, desmosomes. These junctions peculiarly interact with the cellular cytoskeleton and regulate paracellular permeability [28].

The lamina propria must also be mentioned, which includes B and T lymphocytes and phagocytes, is in the other intestinal wall layers, and is responsible for adaptive immunity [13,14]. Interestingly, gut microbes continuously communicate with their host and maintain their homeostasis and specifically natural and adaptive immunity within the intestinal lymphoid tissues [29]. Therefore, pattern recognition receptors (PRRs) are superficialized on dendritic cells, macrophages, monocytes, neutrophils, and epithelial cells, and they can detect pathogen-associated molecular patterns (PAMPs), microbe-associated molecular patterns (MAMPs), and damage-associated molecular patterns (DAMPs) from the surface and luminal gut microbiota. Consequently, the translocation of bacteria and/or their particles/products across the intestinal enterocytes’ barrier is selectively prevented [17].

#### 3.1.2. Gut Microbiota Dysbiosis and MASLD

Non-alcoholic fatty liver disease (NAFLD) is the most diffused chronic liver disease, characterized by excessive hepatic storage of triglycerides in patients with “no or little alcohol consumption” [2]. The term NAFLD was reclassified first as metabolic dysfunction-associated fatty liver disease (MAFLD) and then as metabolic dysfunction-associated steatotic liver disease (MASLD) to avoid the bias arising from the stigma of the terms “non-alcoholic” and “fatty” [2,30]. Thus, patients with MASLD have a micro-inflammatory status, impaired intestinal permeability, increased visceral obesity, and gut dysbiosis [31].

In NAFLD patients, gut dysbiosis is characterized by a reduced Bacteroidetes/Firmicutes ratio, where the growth of noxious genera and a relative abundance of alcohol-producing bacteria are observed [32]. In detail, NAFLD can show decreased abundance of *Bacteroidetes* and *Ruminococcaceae* and increased abundance of *Lactobacillaceae*, *Veillonellaceae*, and *Dorea* [33] and increased abundance of the genus *Lactobacillus* and the family *Lactobacillaceae* and lower abundance of *Bacteroidetes* and *Firmicutes* and of *Ruminococcus*, *Faecalibacterium prausnitzii*, and *Coprococcus*. Importantly, these characteristics are typical of both liver steatosis and of steatohepatitis patients. They are not affected by BMI or severity of insulin resistance [26,27]. In addition, NAFLD patients have an increased abundance of *Lactobacillus* species and the *Lachnospiraceae* genus (e.g., *Dorea*, *Robinsoniella*, and *Roseburia)* and a decreased abundance of *Oscillibacte* (particularly the *Firmicutes* phylum and the *Ruminococcaceae* genus) compared to healthy subjects [34]. More precisely, an updated systematic review and meta-analysis of data revealed that NAFLD patients have similar abundances of *Bacteroides*, *Bifidobacterium*, *Blautia*, *Clostridium*, *Dorea*, *Lactobacillus*, *Parabacteroides*, and *Roseburia* compared to healthy controls. However, they have a higher representation of *Escherichia*, *Prevotella*, and *Streptococcus* and a decreased abundance of *Coprococcus*, *Faecalibacterium*, and *Ruminococcus* [35]. In 25 NAFLD patients, a higher concentration of the *Proteobacteria* and *Fusobacteria* phyla and a lower concentration of *Prevotella* compared to healthy subjects was found [36]. Further, Korean NAFLD patients had an increased abundance of the phylum *Proteobacteria*, the family *Enterobactereriaceae*, and the genus *Citrobacter* and a decreased abundance of the genus *Faecalibacterium* compared to healthy subjects. Of mention, NAFLD patients had a decreased abundance of butyrate-producing bacteria and an increased abundance of ethanol-producing bacterial strains [37].

In 205 NAFLD (n = 205) and 943 non-NAFLD subjects, gut dysbiosis was examined excluding the biasing impact of obesity and sex: the family *Ruminococcaceae* and the genus *Faecalibacterium* had significantly lower representation in NAFLD vs. non-NAFLD subjects [38]. In 766 NAFLD patients, the gut microbiota showed a lower richness because of a high abundance of *Fusobacteria* and a low abundance of the genera *Oscillospira* and *Ruminococcus* of the family *Ruminococcaceae* and the genus *Coprococcus* of the family *Lachnospiraceae* compared to healthy controls [39].

From a functional point of view, in children with MASLD, gut dysbiosis is associated with a higher degree of ingested carbohydrate oxidation compared to healthy children [40]. More in detail, children with NAFLD and also those with NASH showed a significant decline in microbial α-diversity, defined by a reduction in the number of different microbial taxa without the loss of metabolic patterns. The study also found a greater variation in β-diversity (the dissimilarity or distance between microbiome pairs) [21]. In particular, the increased concentration of *Prevotella copri* was associated with NAFLD development. Interestingly, NAFLD severity was associated with a higher expression of genes encoding lipopolysaccharides (LPSs) and the flagellar apparatus, antigens linked to a micro-inflammatory state [41]. Indeed, LPSs can activate toll-like receptor 4 (TLR4) and the innate immunity process. LPSs are part of the cell walls of Gram-negative bacteria like the phylum *Proteobacteria* and the genera *Bacteroides* and *Prevotella*. Peculiarly, TLR4 activation triggers Th17 cell development from naïve CD4 T cells. In this regard, Th17 cells are elevated in NAFLD and in obesity patients showing NASH evolution [42]. Further, flagellin also triggers the innate immune response via toll-like receptor 5 (TLR5) and the nucleotide-binding oligomerization domain (NOD)-like receptor-mediated inflammasome [22]. Hepatotoxic gut microbiome metabolites (trimethylamine, ammonia, trimethylamine-N-oxide, and endogenous alcohol) are transported to the organ through portal circulation [43,44], leading to the activation and release of proinflammatory cytokines and nitric oxide by hepatic Kupffer cells in this inflammatory environment [25].

It is interesting to examine the interaction between MASLD and obesity and their impact on gut dysbiosis. In Asia, Lee et al. distinguished patients with NAFLD/MASLD and obesity from non-obese NAFLD patients. Specifically, NAFLD/MASLD and obesity patients had a lower abundance of *Ruminococcaceae*, an increased abundance of *Veillonellaceae*, and, in general, low microbial diversity compared to non-obese patients. This gut dysbiosis was significantly associated with more severe liver fibrosis [45].

The gut microbiota also accounts for viruses and fungi in addition to protea, yeasts, and archaea. Intestinal virus and fungi communities show differences among patients with NAFLD/MASLD [46,47]. Lang et al. found that NAFLD/MASLD patients with an increased NAS (NAFLD activity score) have decreased bacteriophage diversity compared to patients with a low NAS [35]. Further, Demir et al. found that non-obese patients with more severe forms of NAFLD/MASLD have a different fecal mycobiome compared to patients with obesity with less severe NAFLD/MASLD forms [36] (Table 1).

In conclusion, we can assume that the evidence summarized in Table 1 mainly resembles the gut bacterial dysbiosis typical of MASLD and distinguishing its stages (namely MASLD vs. MASH patients). The uniformity of study methods confirms the strength of the evidence. Obesity seems to affect the gut dysbiosis of MASLD vs. cirrhotic patients.

#### 3.1.3. Microbiota and “Gut–Liver Axis”

The gut–liver axis is a physiopathological model describing the interplay between the gut wall and the liver through porto-systemic circulation, with the gut microbiota being the main actor [55]. Intestinal epithelial cells are connected by dynamic tight junctions (TJs). The main proteins belonging to TJs are claudins, occludins, zonula occludens-1, and junctional adhesion molecules (JAMs) [7,56,57]. TJs maintain the integrity of the gut epithelial barrier. In detail, their 11 angstroms of width do not allow contact between the intestinal epithelial cells (IECs) and microbial pathogens. On the other hand, they allow various nutrients to enter the wall [7,58]. This limits the entry of pathogens via IgA secreted by plasma cells. Moreover, interleukin-23 (IL-23) release induces the activation of group 3 innate lymphoid cells producing IL-22. This promotes the production of antimicrobial peptides from Paneth cells and IECs. Furthermore, portal vein blood enters the liver sinusoids, whose endothelial cells activate the Kupffer cells that translocate into the periportal area and protect the liver from pathogens and gut-derived toxins (e.g., trimethylamine (TMA), p-cresol (PC), and H2S) [7,45,59]. Interestingly, the liver parenchyma harbors fewer T cells, which are less protective against pathogens/antigens/toxics compared intestinal intra-epithelial ones.

Indeed, the “gut–liver” axis can be considered according to a bidirectional reading style. Thus, the “liver–gut” axis has several actors. The bile composition accounts for bile acids (BAs), IgA, antimicrobial peptides, and bicarbonates and has a deep host-defending feature. BAs can be classified as primary, which may be conjugated with glycine/taurine by hepatic cells and secreted in bile by the bile salt export pump (BSEP). Secondary BAs are de-conjugated by the microbiota within the gut lumen [46].

The main bile acid receptors (namely the Farnesoid X-activated receptor (FXR) and G protein-coupled bile acid receptor (GPBAR-1)) are in the gastrointestinal tract (e.g., the liver and intestinal wall) [46]. Of mention, BAs have detergent properties and activate the FXR. Both these actions modulate the gut microbiota composition. Conversely, *Bacteroidetes*, *Lactobacillus*, *Bifidobacterium*, and *Clostridium* operate the de-conjugation of BAs through their production of bile salt hydrolase (BSH) [46,60].

Focusing on FXR agonism, BAs are the endogenous ligand of the FXR. In detail, chenodeoxycholic acid is the main agonist of the FXR. The stimulation of the FXR regulates the BA composition and the transcription of factors associated with lipogenesis, inflammation, and fibrosis. They are crucial in NAFLD/MASLD pathophysiology [46,47]. More in detail, gut dysbiosis finely interacts with bile acids in MASLD patients. In fact, biopsy-proven NASH patients have higher fasting and post-prandial plasmatic concentrations of secondary hydrophobic and cytotoxic deoxycholic acid and lithocholic acid [61]. Secondary bile acids can affect enterocytes’ integrity and shape gut microbiota dysbiosis [27]. For all these reasons, bile acids’ role as hormone-like agonists is affected by gut dysbiosis interaction. In NAFLD patients, the antagonistic FXR effect of deoxycholic acid is upregulated and the agonistic effect of chenodeoxycholic acid is downregulated. The latter follows the plasmatic concentrations of the fibroblast growth factor (FGF19). Thus, there is altered FXR and fibroblast growth factor receptor 4 (FGFR4) signaling [62]. Within this subset of findings, NASH patients exhibited increased concentrations of total fecal bile acid and particularly of primary cholic and chenodeoxycholic acid. Importantly, these concentrations have been associated with a decreased abundance of *Bacteroidetes* and *Clostridium leptum* [28]. Interestingly, the FXR also regulates the intestinal permeability of TJs. In detail, FXR agonism results in the secretion of antimicrobial peptides by IECs. It is important to point out that FXR location conditions its actions. In fact, the FXR located in the ileum participates in the FXR/FGF15/FGF19 pathway (fibroblast growth factor 15 in rodents and fibroblast growth factor 19 in humans) [63]. Altogether, the evidence shows that FXR agonism within the liver could prevent de novo lipogenesis, inflammation, and fibrosis in the frame of NAFLD/MASLD physiopathology [46,47,48].

When gut dysbiosis occurs, the gut–liver axis becomes unbalanced. In fact, the intestinal wall histology of NAFLD patients shows impaired gut permeability and that tight junctions are widened. Furthermore, microvilli architecture is altered because of the decreased levels of the occludin protein compared to that in healthy subjects [6,7]. Thus, gut dysbiosis can be the consequence of the reduction in TJs observed in mice fed a high-fat diet (HFD). In particular, the reduction in TJs is associated with increased intestinal permeability to PAMPs in the frame of a “leaky gut” [46,47,48]. Indeed, a Gram-negative bacterial lipopolysaccharide (LPS) passes through the leaky gut to the portal vein and activates TLRs 4 and 9. The latter activates NF-kB and the inflammasome nucleotide-binding domain, leucine-rich-containing family, and pyrin domain-containing-3 (NLRP3), resulting in the various degrees of hepatic inflammation observed in NASH/MASH patients [64].

The macronutrient choline is metabolized into the liver to phosphatidylcholine, involved in the synthesis of very-low-density lipoproteins (VLDLs). They prevent intrahepatic triglyceride accumulation and are protective against NAFLD [65]. The dietary precursors of trimethylamine (TMA) are mainly choline, phosphatidylcholine, betaine, and L-carnitine, from eggs, red meat, and fish [66]. Indeed, choline deficiency is associated with liver steatosis and oxidative stress [67]. Subsequently, mice fed on a high-fat diet receiving trimethylamine N-oxide (TMAO) showed liver triglyceride storage, lipogenesis, and increased bile acid synthesis, especially those with FXR-antagonistic activity [47]. In fact, in patients with obesity and type 2 diabetes (T2DM), increasing plasmatic concentrations of TMAO, choline, and secondary bile acids dampen the risk of NAFLD and NASH [47]. Intriguingly, the taxa *Erysipelotrichia* can turn choline into TMA, decreasing its bioavailability and increasing its conversion to TMAO and its steatogenic effects [68].

In addition to TMA and LPS levels, an increased plasma bacterial ammonia concentration shows a significant correlation with NAFLD/MASLD prevalence. In fact, ammonia is produced by bacterial fermentation of ingested proteins. The final products of the fermentation are ammonia and branched-chain fatty acids (BCFAs). Therefore, increased plasmatic levels of ammonia and of BCFAs are involved in the development and progression of NAFLD/MASLD [69].

**Figure 1:**
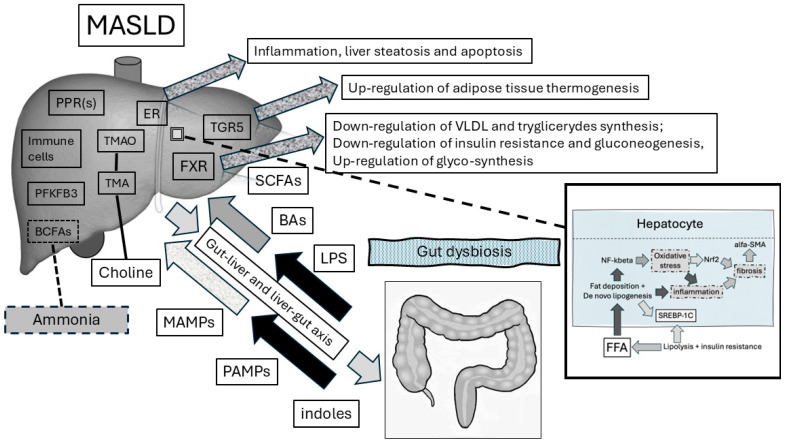
Interaction between gut dysbiosis and the liver in MASLD pathophysiology. The gut–liver axis is a bidirectional functional model explaining the physiologic relationship between the gut microbiome and the liver. In detail, gut dysbiosis components translocate into the liver through impaired intestinal permeability. In further detail, PAMPs (e.g., LPS) activate the innate immune system response. PAMPs sustain chronic low-grade inflammation based on the agonism of pattern recognition receptors (PPRs, namely TLRs). BAs are signals mediating the shift towards suppressed lipogenesis, reduced gluconeogenesis, and increased insulin sensitivity. These actions result from the activation of nuclear receptors (e.g., FXR). In addition, BAs bind to G protein-coupled bile acid receptors in adipose tissue for the maintenance of energy metabolic homeostasis. Within the liver, TMA is converted to TMAO. The latter binds to endoplasmic reticulum oxidative stress enzymes and induces cellular apoptosis and inflammation. Conversely, SCFAs maintain lower degrees of inflammation. Similarly, indoles upregulate the anti-inflammatory 6-phosphofructo-2-kinase/fructose-2,6-biphosphatase 3 (PFKFB3) gene expression and, on the other hand, reduce the expression of genes responsible for lipogenesis (e.g., Srebf1, ACCA1, and PPARγ). Indeed, both indoles and SCFAs restrain the entry of LPS into the blood torrent to the liver and re-establish intestinal permeability. We show also the role of oxidative stress in the pathophysiology of hepatocyte damage in the bottom-right box figure: free fatty acids (from lipolysis and favored synthesis by insulin resistance) enter the hepatic cell with fat deposition and liver steatosis takes place because of de novo lipogenesis. Fat accumulation is favored by the SREBP1c cascade. FFAs induce the inflammatory response via the NF-kB pathway and mitochondrial oxidative stress that acts on the NRF2 pathway. Liver fibrosis activates the alpha-SMA pathway that participates in the fibrotic process. List of abbreviations: alpha-SMA—alpha-smooth muscle actin; BA—bile acid; FXR—Farnesoid X receptor; LPS—lipopolysaccharide; NF-kB—nuclear factor kappa β; NRF2—nuclear factor erythroid 2-related factor 2; PAMPs—pathogen-associated molecular patterns; PPAR—peroxisome proliferator-activated receptors; SCFAs—short-chain fatty acids; SREBP1c—sterol regulatory element-binding protein 1c; TGR5—bile acid G protein-coupled receptor 5; TLR—toll-like receptors; TMA—trimethylamine; TMAO—trimethylamine oxide; ER—endoplasmic reticulum; SREBF1—sterol regulatory element-binding transcription factor 1; ACCA1—acetyl-coenzyme A carboxylase carboxyl transferase subunit alpha 1; BCFAs—branched-chain fatty acids.

Figure 1 depicts the actors of the gut–liver axis and their interactions in physiologic conditions and, when gut dysbiosis occurs, in the pathophysiology of NAFLD/MASLD.

#### 3.1.4. Oxidative Stress Within the Gut—Liver Axis Model for MASLD

Generally, oxidative stress and inflammation finely interact through a positive feedback loop. The result is liver fibrosis. In fact, the accumulation of ROS and lipid peroxidation (LPO) products leads to hepatocyte apoptosis/necrosis. The latter activates HSCs [18]. These cells may promote fibrosis in the attempt to heal liver damage [18]. In detail, the ROS-mediated activation of HSCs is followed by α-smooth muscle actin (α-SMA), vimentin (VIM), and collagen storage in the extracellular matrices [18,70]. In addition, liver fibrosis is also generated by the upregulation of the malondialdehyde/**4**-hydroxynonenal (MDA/**4**-HNE) pathway. Particularly, profibrogenic matrix metalloproteinase-2 (MMP2) upregulation and matrix metalloproteinase-1 (MMP1) remodel the hepatic extracellular matrix [18,68].

In alcohol-induced ROS-mediated liver fibrosis, acetaldehyde and other reactive aldehydes stimulate the expression of fibrogenic transforming growth factor-β (TGF-β), which results in the production of collagen and α-SMA [18,71]. Other post-translational modifications (namely acetylation and methylation) are involved in the progression of fibrosis in ALD [18,72]. Indeed, tissue inflammation is worsened by impaired intestinal permeability [18,53]. Specifically, hyper-endotoxemia interacts with toll-like receptor 4 (TLR4) in Kupffer cells and produces NADPH-oxidase (NOX)-dependent ROS and HSCs [18,53]. Additionally, nuclear factor erythroid 2-related factor (Nrf2) and its downstream antioxidant enzymes and proteins may be exhausted, resulting in lowered antioxidant levels [18,53].

As the pathophysiology of MASLD has several similarities with ALD, altered lipid metabolism contributes to lipotoxicity and peroxidation with endoplasmic reticulum (ER) stress, mitochondrial dysfunction, and finally hepatocyte damage [18,53,73]. Firstly, fat accumulation leads to steatosis, and then mitochondrial respiration increases and ROS production rises, activating antioxidant responses [53,74]. Stored free fatty acids (FFAs) compromise mitophagy responses through the activation of C-Jun N-terminal protein kinase (JNK)-dependent apoptosis [53,71]. In MASH, inflammation and oxidative stress show a vicious and positive feedback loop profile: hepatocyte apoptosis can impair cellular respiration, mitophagy, and antioxidant response. When chronic inflammation, oxidative stress, and hepatocyte apoptosis together trigger Kupffer cell and HSC activation and neutrophil infiltration, fibrosis takes place [53,71].

Thus, despite the shared steps of pathophysiology involving ALD and MASLD, the “multiple hit” hypothesis seems to better explain the features of MASLD/MASH. Specifically, increased fat accumulation can be considered the “first hit”, followed by oxidative stress as the “second hit” [75]. Specifically, elevated FFAs are more hepatotoxic than triglyceride accumulation. In fact, FFAs cause JNK-mediated hepatocyte apoptosis with a subsequent cytokine storm and MASH progression [73]. In addition, FFA accumulation leads to oxidative stress and causes an NF-κB-related inflammatory cascade and further activation of HSCs [73]. More in detail, FFAs and ROS-induced liver cell apoptosis are mediated by the B-cell lymphoma (Bcl)-2 and Bcl-2 Associated X-protein (Bax)-related mitochondrial functioning derangement. Indeed, nicotinamide adenine dinucleotide phosphate oxidase 4 (NOX4) and ethanol-inducible cytochrome P450-2E1 (CYP2E1) can oxidize long-chain FFAs, resulting in uncoupled electrons leaking from the mitochondrial electron transport chain [68]. Moreover, after FFA oxidation in peroxisomes and the ER, oxidative stress activates the Bax/Bcl-2 complex through fork head box (Fox)Oa3 and JNK [73,76]. Further, Bax release from Bcl-2 can induce a mitochondrial permeability transition (MPT) following oxidative stress that results in cytochrome c release and the activation of caspase-mediated apoptosis [74]. In addition, activated JNK stimulates Bax phosphorylation and its translocation to mitochondria, with altered mitochondrial permeability and hepatocyte apoptosis [74,77].

Cholesterol metabolism dysregulation significantly characterizes the progression of MASLD. Indeed, in the frame of oxidative physiology maintenance, the cholesterol to bile acid ratio is vital in supporting the homeostatic redox environment of HSCs [78]. In this regard, cholesterol selectively induces oxidative stress and reduces liver fibrosis through HSC apoptosis induction [76]. In fact, a good cholesterol index (a higher concentration of high-density lipoprotein (HDL) cholesterol and a lower concentration of low-density lipoprotein (LDL) cholesterol) can significantly protect from MASLD development because HDLs allow the liver filtration of LDLs and other fats and their excretion rather than storage in target-damage organs like blood vessels and tissues (e.g., the liver) [76,79].

More in detail, elevated hepatic free cholesterol has been described in MASH patients compared to healthy subjects [80]. In fact, high-fat, high-cholesterol diets are significantly associated with MASH development compared with high-fat, low-cholesterol regimens [81]. Furthermore, cholesterol-enriched oxidate-LDL uptake by hepatic Kupffer cells fosters inflammation in MASH patients [82]. This pathophysiological link is possible because of the lysosomal cholesterol crystal formation within macrophages (a DAMP signal activating the inflammasome) [83].

Fluctuations in cellular cholesterol concentrations are due to sterol regulatory element-binding protein-2 (SREBP-2) gene expression. In fact, the gene activates 3-hydroxy-3-methyl-glutaryl-coenzyme A reductase (HMGCR), responsible for cholesterol synthesis [84]. Further, comparing MASH and MASLD patients, a significantly higher expression of SREBP-2 was shown in MASH patients only [85]. In Alms1 mutant (foz/foz) mice fed with a high-fat diet, there was significant overexpression of SREBP-2 compared to wild-type mice [80]. Interestingly, the mice had high concentrations of free cholesterol due to elevated LDL-receptor (LDL-R)-mediated uptake and reduced bile synthesis and liver secretion [86]. On the other hand, patients administered with statins showed significantly reduced SREBP2 and HMGCR mRNA expression in liver samples compared to non-treated MASLD/MASH patients. Furthermore, in diabetic and obese mice, the administration of atorvastatin and ezetimibe, one reducing cholesterol production and the other its uptake by HMGCR inhibition and the Niemann–Pick C1-like 1 (NPC1L1) proteins, significantly reversed liver fibrosis of MASH mice [87].

The altered balance of bile acids’ synthesis (via cholesterol-7α-hydroxylase (CYP7A1)), of their transportation (mainly through the ATP-binding cassette transporters (ABCG)5/8/B11 and Solute Carrier Family 10 Member 1 (SLC10A1)), and of their signaling (regulated by the nuclear Farnesoid X receptor (FXR) and the liver X receptor (LXR)) contributes to the progression of hepatic steatotic profibrogenic damage [62,88]. Intriguingly, when steroidogenic acute regulatory protein (StarD1) is overexpressed in response to Western-diet mice feeding, bile acid synthesis significantly rises. Therefore, there is reduced hepatic lipid content (precisely, triacylglycerol, total cholesterol, and FFAs) [89]. Indeed, oxysterol 7α-hydroxylase (CYP7B1, responsible for the oxysterol to bile acid conversion) is significantly inhibited by increased StarD1 activity [89].

A dietary imbalance with excessive omega-6 polyunsaturated fatty acids (PUFAs) and insufficient omega-3 PUFAs has been associated with NAFLD development [90]. In fact, evidence from the literature has shown that NAFLD patients have a higher n-6/n-3 ratio and a lower PUFA content. Omega-3 PUFAs are represented by docosapentaenoic acid (DPA), eicosapentaenoic acid (EPA), stearidonic acid (SDA), docosahexaenoic acid (DHA), and alpha-linolenic acid (α-ALA) [91]. Only a small fraction of dietary ALA is converted to DHA, which is the most prevalent omega-3 PUFA stored in the liver [81]. Following that, oxidative stress and free radical production leads to hepatic desaturase inactivation and omega-3 PUFA depletion by reduced synthesis [92]. In the case of lowered desaturase activity within the liver (namely obesity-induced NAFLD), studies in HFD-fed mice showed upregulation of the SREBP-1c-dependent mRNA expression of fatty acid desaturase (FADS)1 and FADS2 [93]. Importantly, this can be considered a feature of the adaptive response to the reduced content of hepatic omega-3 PUFAs. This allows SREBP-1c activation when insulin resistance is elicited [94].

On the other hand, increased oxidative stress may induce de novo lipogenesis through upregulation of sterol regulatory element-binding protein-1 (SREBP-1) and mitochondrial dysfunction in MASLD patients [76,77]. In MASH, ROS are generated by mitochondrial electron leakage, pro-oxidative enzyme activation (namely CYP2E1 and NOX4), iron accumulation, Fenton reaction metabolism, and antioxidant depletion [76,77]. Thus, we can hypothesize that oxidative and lipotoxic stress can be buffered by several antioxidants. Therefore, the Nrf2-induced antioxidant response element (ARE) pathway seems to be crucial in MASLD prevention. In detail, the transcription factor Nrf2 physiologically binds to Kelch-like ECH-associated protein (Keap)1. During oxidative stress occurrence, ROS oxidizes Keap1, which is cleared up through ubiquitin-dependent degradation and releases Nrf2. Upon Nrf2 release, it is bound to ARE in the cellular nucleus. Interestingly, activation of the Nrf2/ARE pathway upregulates the transcription of several antioxidant enzymes (namely heme-oxygenase (HO)-1 and Nicotinamide Adenine Dinucleotide Phosphate Hydrogen (NADPH)-dependent quinone reductase) and reduces glutathione (GSH) synthesis enzymes (e.g., glutathione reductase (GR) and glutamate-cysteine ligase modifier subunit (GCLM)) [76,77].

Several antioxidants have been used in animal and clinical research to treat MASLD: vitamins E and C, caffeine, and coffee polyphenols [95]. The latter are mainly derived from green tea. After coffee, green tea is the second most consumed beverage in the world. Its major polyphenols are represented by flavanols, mainly catechins. Epigallocatechin-3-gallate (EGCG) is the most abundant catechin found in tea. It represents 50–75% of the catechins [96], with beneficial effects in humans. For example, Bose et al. found reduced insulin resistance and liver steatosis in mice fed a Western diet with an EGCG add-on [97]. Interestingly, Kuzu et al. showed that EGCG administration significantly reduces liver steatosis and inflammation in rats fed a high-fat diet. These findings were obtained through lipid peroxidation and CYP2E1 expression reduction and the restoration of GSH levels [98]. Moreover, EGCG was shown to inhibit HSC activation in several in vitro studies with human-derived HSCs [99]. Further, Xiao et al. found that intraperitoneal EGCG administration in a rodent model of NASH was able to reduce liver fibrosis through the suppression of oxidative stress and inhibition of NFkB, Akt, and TGF/Suppressor of Mothers against Decapentaplegic (SMAD) signaling [100]. Thus, the use of EGCG in NASH and, lately, MASH patients is warranted in future clinical trials.

Data from a recent systematic review of the literature show that EGCG administration is also able to significantly decrease total cholesterol, triglycerides, and low-density lipoprotein (LDL) concentrations [101]. Lee et al. found that EGCG has a dose-dependent effect on adipocyte differentiation genes. In particular, mRNA expression of PPAR, C/enhancer binding protein (EBP)-, lipoprotein lipase (LPL), and fatty acid synthase (FAS) were markedly decreased upon EGCG treatment. This was significantly correlated with the reduction in adipose tissue deposition [102]. Li et al. showed that EGCG supplementation can activate silent information regulator-1 (SIRT-1) that increases the expression of fork head box protein O1 (FOXO1) and, conversely, decreases the expression of SREBP-2. Subsequently, increased FOXO1 expression increases antioxidant catalase activity [29].

Very interestingly and finally, mitochondria-targeting synthetic and natural antioxidants (namely melatonin) have a promising potential to treat, prevent, and reverse MASLD progression [78,103].

### 3.2. Gut Microbiota Modulation for MASLD Treatment

#### 3.2.1. Diet in MASLD Treatment

The efficacy of diet in reshaping the gut microbiota is based on evidence from animal studies. In mice, a high-fat diet was associated with a nearly 60% reshaping of the gut microbiota [104]. From a clinical and therapeutic point of view on MASLD, the Mediterranean diet shows promising findings. This type of diet, high in vegetable and fruit intake, is enriched with antioxidants (e.g., flavonoids and terpenes). Twenty individuals with obesity were administered a Mediterranean diet and a low-fat, high-complex-carbohydrate diet (LFHCC) for one year. Interestingly, there was a significant shift in their fecal microbiota composition: an increased concentration of the *Roseburia* genus and *F. prausnitzii* [105]. Liver steatosis and type 2 diabetes were prevented accordingly. Other dietetic regimens mimicking the Mediterranean diet mainly consist of vegetable-based diets. These regimes are rich in SCFAs, and the modulated gut microbiota of those who adhere to these diets has been shown to have an abundance of *Prevotella* and fiber-degrading *Firmicutes*. Oppositely, subjects with scarce adherence to the Mediterranean diet have higher urinary trimethylamine oxide levels [106].

Diet can induce one-day-like quick shifts in gut microbiota composition. For example, men exposed to a quick increase in the consumption of animal-derived products had a similar increase in the relative abundance of *Alistipes*, *Bilophila*, and *Bacteroides* (specifically bile-tolerant bacteria) and a decrease in the abundance of *Firmicutes Roseburia*, *Eubacterium rectale*, and *Ruminococcus bromii*, and were capable of metabolizing plant-derived polysaccharides [107].

In a study of men at risk of developing of metabolic syndrome, the subjects were randomized to a high-saturated-fat diet, high-monounsaturated-fatty-acid (MUFA)/high-glycemic-index (GI) diet, high-MUFA/low-GI diet, high-carbohydrate (CHO)/high-GI diet, and high-CHO/low-GI diet. Typically, fecal *Bacteroides* abundance rose after consumption of the high-CHO/high-GI diet, and the abundance of *F. prausnitzii* rose after the high-CHO/low-GI diet. On the other hand, subjects administered with the high-MUFA/high-GI and high-MUFA/low-GI diets showed reduced total bacteria count and increased fecal SCFA concentration. This result can be explained by the response of the gut microbiota to excessive energy intake [108]. The evidence was confirmed by a recent meta-analysis [109].

Spahis et al. supplemented 11 young French-Canadian males with NAFLD with 2 g/day of fish oil (1.2 g of EPA + DHA) for 6 months. This brought a significant increase in red blood cell EPA and DHA levels and a decrease in oxidative stress markers. Both resulted in reduced hepatic steatosis [110]. Subsequently, 30 young French-Canadian NAFLD males underwent baseline dosing of plasma FA. The latter was characterized by high n-6 PUFA, saturated FA, and monounsaturated FA and lower delta desaturase 5 (D5D) and higher D6D activity. Three months of fish oil supplementation resulted in an improved omega-3 PUFA profile, with a significant increase in EPA and DHA and D5D activity. On the other hand, MUFA and the omega-6/omega-3 ratio significantly decreased [111]. Pacifico et al. evaluated the effect of six months of DHA supplementation (250 mg/day) in 25 biopsy-proven NAFLD children. Significantly, there was an almost 50% reduction in liver fat deposition shown in magnetic resonance imaging. There were also reduced fasting insulin and triglyceride plasmatic levels [112]. Long-term fish oil supplementation (503 mg/day DHA and 103 mg/day EPA) in adult NAFLD patients resulted in increased red blood cell DHA concentrations and an increased omega-3 index (namely EPA + DHA) and significantly reduced liver fibrosis [113]. In biopsy-proven NASH patients, a six-month treatment of omega-3 PUFAs (64% ALA, 16% EPA, and 21% DHA) 0.945 g per day resulted in increased plasma ALA and EPA concentrations, a decreased triglyceride concentration, and an improvement/stabilization of the steatohepatitis activity score [114].

Pansevich et al. compared the effect of a soy- vs. dairy-protein-based diet on the gut microbiota and liver steatosis of hyperphagic Otsuka Long-Evans Tokushima Fatty (OLETF) rats. They were randomized to a Western diet containing a milk protein isolate (MPI), a soy protein isolate (SPI), or 50:50 MPI/SPI (MS) (n = 9–10/group) for 16 weeks. Interestingly, the SPI reduced fat mass compared to the MS but not compared to the MPI. Consequently, histologically determined liver steatosis was lower after SPI compared to MPI or MS administration. There was a reduced hepatic concentration of diacylglycerols after the SPI compared to the MPI. This was associated with lower hepatic de novo lipogenesis compared other diets. A fecal bacteria 16S rRNA analysis showed the SPI being associated with an increased abundance of *Lactobacillus* and a decreased abundance of *Blautia* and *Lachnospiraceae.* This resulted in a reduced concentration of fecal secondary bile acids in the SPI-fed rats. Finally, the SPI and MS diet administrations were associated with higher hepatic FXR, FGFR4, hepatocyte nuclear factor alpha (HNF4)a, HMGC reductase, and synthase mRNA expression compared to the MPI [115].

Green tea catechins are supplements that have been widely studied over the past two decades for NAFLD and lately for MASLD. Green tea flavonoids have significant anti-inflammatory and antioxidative actions [116]. In particular, EGCG has a potential therapeutic profile in MASLD subjects. In fact, the reviewed rodent and human studies support its clinical use. In further detail, at least 12 weeks of a 300–600 mg/day administration of EGCG is associated with an improved lipid profile, a reduced oxidative status, and improved liver damage [117]. Randomized placebo-controlled human trials are needed to confirm the mechanistic data.

#### 3.2.2. Prebiotics, Probiotics, and Symbiotics in MASLD Treatment

Gut microbial composition can be restored to a healthy status by indigestible carbohydrates, namely prebiotics, microorganisms with beneficial effects for host health, namely probiotics, or components of probiotics, namely postbiotics [118]. Thus, the gut microbiota shows increased gut microbial diversity, and treated subjects show reduced LPS activation and improved insulin sensitivity [119].

There are few data on the use of prebiotics in NAFLD/MASLD experimental models and humans. However, a meta-analysis on 25 studies testing the use of prebiotics in obesity found they had a significant impact on obesity and fat deposition [120]. Interestingly, the association between prebiotics and phytochemicals shows a promising additive effect on gut microbiota remodulation. In detail, alpha-galacto-oligosaccharides from legumes can reduce nutrient ingestion and improve plasma lipids, free fatty acids, and triglyceride liver storage, resulting in reduced liver steatosis in high-fat-diet-fed mice [121]. The plant flavanol quercetin (specifically, the flavonoid group of polyphenols) can increase the intestinal abundance of *Akkermansia* and, subsequently, can reduce hepatic fat accumulation [122]. Moreover, mice undergoing dietary supplementation with curcumin exhibit a taxonomic shift in gut microbial composition and reduced liver steatosis associated with decreased fat accumulation and improved intestinal permeability [123].

In vitro studies on *Lactobacillus brevis* showed its capability to restore intestinal permeability (assessed with electrical resistance) after cytokine-induced barrier impairment. In detail, the effect is explained by the production of anti-inflammatory IL-10 and alkaline phosphatase that regulate endotoxin passage in the gut [124].

In obese diabetic mice, *Bifidobacterium animalis* ssp. *lactis* 420 significantly decreased high-fat-diet-induced body weight increase and diabetes occurrence. Interestingly, the probiotic reduced plasma LPS levels, liver inflammation, and *E. coli* adhesion in the distal colon [125]. In addition, in a three-month randomized, double-blind, placebo-controlled study, Szulinska et al. found a multispecies probiotic to improve glucose metabolism, lipid asset, waist circumference, visceral fat, including liver steatosis, and LPS concentration in postmenopausal women with obesity [119]. Mechanistically, in a randomized controlled trial in diabetic patients, 4-week supplementation with *Lactobacillus acidophilus NCFM^®^* improved insulin sensitivity through the reduction in LPS levels and agonism of TLRs and the production of cytokines. Importantly, the systemic inflammatory response was not modulated by the probiotic [126]. Furthermore, the use of a double-strain probiotic with *Lactobacillus* and *Bifidobacteria* led to reduced levels of serum alanine aminotransferase in NAFLD patients [127]. Similar results were obtained with the administration of *Streptococcus thermophiles* [128]. *Akkermansia muciniphila* is a metabolically acting probiotic strain whose abundance, in turn, can be increased by dietary polyphenols, flavonoids, and alkaloids [129]. However, we must recognize that a rodent study showed that the increased abundance of *Akkermansia muciniphila genotype 1* was correlated with impaired lipid metabolism and a hyper-inflammatory state [130]. Following this strain investigation, in humans with obesity vs. non-obese insulin-resistant humans, the randomized administration of pasteurized *Akkermansia muciniphila* for three months was only slightly associated with loss of body weight, but improved insulin sensitivity was shown [131]. In 90 subjects with obesity under a 6-week calorie restriction, *Akkermansia muciniphila* supplementation significantly reduced visceral (e.g., liver steatosis) and subcutaneous adiposity [132].

In a mouse model, *Faecalibacterium prausnitzii* supplementation was shown to restore intestinal barrier integrity. Conversely, its reduced abundance in the intestine correlates with an increased inflammatory state and obesity [133].

Finally, *Roseburia* and *Alistipes* have been shown to protect humans from the development of obesity and metabolic disorders [134].

In a mouse model of liver steatosis with probiotics administered for 4 weeks, liver steatosis was significantly reduced [135]. Further, in a rat model of NAFLD on a choline-deficient/L-amino-acid-defined (CDAA) diet, the rats were administered a butyrate-producing probiotic, MIYAIRI 588, and showed significantly lowered hepatic triglyceride storage, insulin resistance, serum endotoxin levels, and hepatic inflammatory blood tests [136]. In humans, a pilot trial with a mixture containing 500 million *Lactobacillus bulgaricus* and *Streptococcus thermophiles*/day was run in NAFLD patients. These patients had improved liver aminotransferases levels [137]. Furthermore, the multi-strain probiotic VSL#3 administered for 4 months was able to decrease liver steatosis in NALFD children, observing a GLP-1 level increase [138].

Horvath et al. evaluated the impact of a 6-month treatment with a multispecies probiotic in combination with a symbiotic on intestinal permeability (specifically determined through zonulin and LPS expression) and gut microbiota composition in a randomized, double-blind, placebo-controlled trial. No significant derangements in taxonomic composition or alpha- or beta-diversity of the microbiome between groups at any time points of follow-up were recorded. Indeed, the decreased expression of zonulin due to the symbiotic led to better intestinal permeability [139]. More investigations on the use of symbiotics are warranted, especially in the field of MASLD (Table 2).

The data presented in Table 2 show how the use of prebiotics in MASLD has little beneficial evidence, perhaps partial and preliminary. On the other hand, animal and human studies on the use of probiotics in MASLD seem more solid and show effective gut microbiota modulation and lowered liver inflammation and hepatic fat deposition. Specifically, different effects depend on certain strain/multi-strain preparations.

#### 3.2.3. Fecal Microbiota Transplantation in MASLD Treatment

Fecal microbiota transplantation (FMT) is defined by the instillation of processed stool bacteria collected from a healthy donor into the intestinal tract of a sick subject. Of mention, the screening of donors is a limiting step to reduce the potential transfer of pathogens from the donor to the recipient [143,144]. According to the novel FDA-approved formulations, there are ingestible capsules and rectal suspensions. FMT was first approved for recurring *Clostridiales difficile* infections [90,91].

FMT can effectively reverse gut dysbiosis from several causes and positively affect metabolism in animal models and humans [145]. In fact, germ-free mice receiving FMT from obese mice gained body weight [146]. The phenotype change depends on the increased expression of genes encoding for enzymes digesting dietary polysaccharides and increased energy harvesting. Similarly, human FMT donors from twins discordant for obesity were instilled into normal weight germ-free mice and resulted in comparable findings [147].

In human interventional studies, metabolic syndrome patients showed improved insulin sensitivity after 6 weeks from FMT administration [148]. However, the results are promising but not uniform: two randomized controlled trials failed to confirm the findings after allogenic FMT from lean donors [149,150]. Indeed, a meta-analysis of randomized controlled trials in metabolic syndrome subjects reinforced the short cycle of FMT efficacy to reduce glucose, insulin, and glycated hemoglobin levels and increase HDL cholesterol vs. a placebo [80].

More accurately, a few trials studied the use of FMT in liver steatosis patients, namely NAFLD/MASLD patients. Patients receiving a 6-week course of FMT from lean, healthy donors showed a tendency toward improvement in hepatic insulin sensitivity and increased butyrate-producing microbial concentration. Butyrate can prevent the translocation of endotoxic bacterial particles and significantly modulates insulin resistance [151]. Importantly, the gut–liver axis seems to be rebalanced by FMT in liver steatosis patients. Six weeks after allogenic FMT, a significant restoring of impaired small intestinal permeability has been described [152] (Table 2).

The FMT studies shown in Table 2 present promising data on the gut dysbiosis modulation and improved intestinal permeability of treated subjects, resulting in MASLD improvement.

## 4. Conclusions and Future Perspectives

The human bacterial gut microbiota has a defined composition that stabilizes after three years of age. Every pathologic condition is associated with a perturbed intestinal microbiota composition named gut “dysbiosis”. Metabolic diseases have a statistically significant association with intestinal dysbiosis. Gut dysbiosis is a characteristic feature of obesity, insulin resistance, and other features of metabolic syndrome.

More in detail, MASLD and its different steatosis/fibrosis stages have different dysbiosis hallmarks. The pathophysiological model of the “gut–liver axis” explains the development of MASLD. There is solid evidence that gut dysbiosis can alter intestinal permeability and allow the passage of antigens that, through the portal circle, reach the liver parenchyma and trigger inflammation. This happens in the frame of preliminary hepatocyte triglyceride storage. Interestingly, the gut–liver axis can also be considered as the “liver–gut axis”. The altered hepatic immune and metabolic environment can maintain intestinal wall leakage, gut dysbiosis, and PAMP passage in a sort of vicious circle.

Thus, diet components, fatty acids, bile and its acids, bacterial components, and antigens can finely interact with intestinal and hepatic receptors and innate and adaptive immunity, leading to micro-inflammation, fat accumulation in the liver, and fibrosis development [153]. Oxidative stress can be the “second hit” in the pathophysiological process of MASLD development, initiated by the fat deposition and inflammatory cascade triggered by the leaky gut and endotoxin passage [73,121]. Oxidative stress can be the target for novel treatments of MASLD, avoiding its progression to MASH. In fact, the oxidative-stress-induced damage of liver cells seems to be the crucial step for MASH development. The use of antioxidants can be beneficial to its reversal. However, evidence from the literature is derived from mechanistic animal and human studies. Polyphenols and cholesterol-lowering drugs have a promising therapeutic profile that deserves multicentric, randomized, placebo-controlled studies. There is an important interaction between cholesterol metabolism, fatty acid oxidation, and oxidative stress that requires dedicated attention from researchers. This understanding can pave the way for a multi-hit curative approach to MASLD.

There are several pieces of evidence regarding the therapeutic and reversal effects of diet, prebiotics, probiotics, and symbiotics on MASLD. There is initial evidence that the Mediterranean diet is effective in reversing gut dysbiosis and reducing the hepatic inflammation responsible for MASLD and MASH pathophysiologic progression. The use of PUFAs has been significantly associated with lower fat deposition in MASLD and MASH patients. Larger future studies are needed to confirm these promising results. The administration of soy proteins has been associated with reduced hepatic lipogenesis and fat deposition and gut microbiota eubiosis re-establishment. These data are few and need new, larger studies to be confirmed.

The use of prebiotics in MASLD is supported by promising animal and only a few human studies. Indeed, the data are not uniform, and gut microbial modulation cannot yet be defined as beneficial for restoring intestinal permeability and reducing hepatic fat deposition. Several human studies support the use of strain/multi-strain probiotic mixtures for MASLD/MASH treatment. Beneficial microbes seem to be able to modulate intestinal microbiota composition, improve intestinal permeability, reduce oxidative stress, and improve glycemic control and visceral fat deposition. Larger, multicentric RCTs are warranted for future research to come.

There is not yet solid evidence on the efficacy of FMT in MASLD reversal/treatment in humans, but promising results have been recorded in the literature [154]. The main effects obtained with FMT are deep gut microbiota modulation with improved nutrient absorption and metabolism, improved intestinal permeability, improved insulin resistance, and lower fat deposition.

Future multi-center RCTs are warranted to confirm the findings on gut microbiota modulation in MASLD, with a special focus on FMT.

In conclusion, the Mediterranean diet as an add-on to PUFAs, probiotics, antioxidants, cholesterol-lowering drugs, and finally FMT seems to be the road for future research on MASLD treatment. This field of investigation is focused on gut microbiota modulation and the reduction in oxidative stress and liver fat deposition in humans.

## Figures and Tables

**Table 1:** Gut dysbiosis and MASLD stages.

Liver Condition	Bacterial Dysbiosis	Reference
MASLD vs. non-MASLD group (90 vs. 90 subjects) undergoing metagenomic shotgun sequencing	Slackia and Dorea formicigenerans ↑ in MASLD subjects Methanobrevibacter and Phascolarctobacterium ↓ in MASLD subjects	[48]
MASLD (n = 12) vs. MASH (n = 18) vs. HV (n = 27) undergoing 16S rRNA analysis	Fusobacteria and Fusobacteriaceae ↑ in MASH subjects	[49]
MASLD (n = 65) vs. HV (n = 76) undergoing 16S rRNA analysis	Collinsella ↑ in MASH subjects	[50]
MASLD (n = 205) vs. HV (n = 669) undergoing 16S rRNA analysis	Ruminococcaceae and Faecalibacterium ↓ in MASLD subjects	[27]
MASLD (n = 472) vs. HV (n = 883) subjects undergoing 16S rRNA analysis	Coprococcus ↓ and Ruminococcus Gnavus ↑ in MASLD subjects	[51]
MASLD (n = 15) vs. MASH (n = 24) vs. HV (n = 28) subjects undergoing 16S rRNA analysis	Ruminococcus, Faecalibacterium prausnitzii, and Coprococcus ↓ in MASLD subjects	[21]
MASLD (n = 30) vs. MASH until LC (n = 26) subjects undergoing 16S rRNA analysis	Bacteroides ↑ as hallmark of MASH diagnosis; Ruminococcus ↑ is an independent hallmark of increasing liver fibrosis	[52]
MASLD (n = 28) vs. MASH (n = 9) with severe liver steatosis undergoing 16S rRNA analysis	Clostridium spp. ↓ according to increasing steatosis stage; Escherichia/Shigella ↑ according to increasing liver fibrosis	[53]
MASH (n = 17) vs. LC (n = 25) and HV (n = 51) subjects undergoing 16S rRNA analysis	Gram-negatives ↑ in MASH subjects and Megasphaera spp. ↑ in LC subjects	[54]

Table legend: ↑—increased abundance; ↓—decreased abundance; MASLD—metabolic-dysfunction-associated fatty liver disease; MASH—metabolic dysfunction-associated steatohepatitis; HV—healthy volunteers; LC—liver cirrhosis.

**Table 2:** Impact of prebiotics, probiotics, symbiotics, and FMT on gut dysbiosis in MASLD.

Liver Condition	Gut Microbiota Treatment	Results	Reference
MASLD patients with diabetes mellitus (n = 60; 15 each arm), RCT	Prebiotics (butyrate and inulin, used alone or in combination vs. placebo)	↑ A. muciniphila upon inulin and butyrate administration; ↓ Kruppel-like factor 5 mRNA expression; ↑ microRNA-375 after butyrate and butyrate + inulin administration	[74]
MASLD patients (n = 24) with and without diabetes mellitus (n = 24) vs. HV	Probiotics	Improved insulin sensitivity	[140]
MASLD patients with diabetes mellitus (n = 70)	Probiotic with *Lactobacillus casei* spp.	↑ Lactobacillus and ↓ total count of fecal bacteria	[141]
MASLD (n = 26) patients with obesity and diabetes mellitus, RCT	Symbiotics	Reduced hip circumference; improved intestinal permeability with ↓ zonulin and LPS expression	[87]
MASLD (n = 63) patients with obesity, RCT	Symbiotics	↓ Firmicutes/Bacteroidetes ratio	[142]
MASLD (n = 18) patients	FMT: 9 undergoing FMT from lean male donors (allogenic group), 9 undergoing self-FMT (autologous group)	↑ Gut microbial diversity with increased abundance of butyrate-producing Roseburia Intestinalis and Eubacterium hallii ↓ Insulin resistance	[102]
MASLD (n = 38) patients	FMT: 26 undergoing FMT from lean male donors (allogenic group), 12 undergoing self-FMT (autologous group)	↑ Insulin sensitivity	[98]
MASLD (n = 30) patients	FMT: 10 undergoing allogenic FMT from lean vegan donors, 10 undergoing autologous FMT	Allogenic FMT patients with fecal gut microbial composition like those of vegan donors	[99]

Table legend: ↑—increased abundance; ↓—decreased abundance; MASLD—metabolic dysfunction-associated fatty liver disease; RCT—randomized clinical trial; LPS—lipopolysaccharide; FMT—fecal microbiota transplantation.

## Data Availability

All the data reviewed in the manuscript can be retrieved from the main medical databases (e.g., PubMed and Medline) and on the website of the most important gastroenterology and hepatology international meetings (e.g., UEGW, DDW, AASLD, and EASL).
